# Toxoplasmosis, leptospirosis and brucellosis in stray dogs housed at the shelter in Umuarama municipality, Paraná, Brazil

**DOI:** 10.1186/1678-9199-19-23

**Published:** 2013-09-25

**Authors:** Márcia Küster de Paula Dreer, Daniela Dib Gonçalves, Isabel Cristina da Silva Caetano, Edson Gerônimo, Paulo Henrique Menegas, Danilo Bergo, Fabiana Maria Ruiz Lopes-Mori, Aline Benitez, Julio Cesar de Freitas, Fernanda Evers, Italmar Teodorico Navarro, Lisiane de Almeida Martins

**Affiliations:** 1 Department of Preventive Veterinary Medicine and Public Health, University of Paraná (UNIPAR) Umuarama, Paraná, Brazil; 2 Department of Preventive Veterinary Medicine, Londrina State University (UEL), Londrina Paraná, Brazil; 3 Department of Preventive Veterinary Medicine and Public Health, University Center Philadelphia (UNIFIL), Londrina, Paraná, Brazil

**Keywords:** *Leptospira* spp, *Brucella abortus*, *Brucella canis*, *Toxoplasma gondii*, Zoonosis

## Abstract

**Background:**

Leptospirosis, toxoplasmosis and brucellosis are diseases with worldwide distribution. Among stray dogs, these zoonoses are facilitated by direct contact with other animal species, by the habit of scavenging garbage and hunting in search of food, drinking standing water, smelling other animals’ urine, licking female genitalia and the sexual act itself. The objective of this study was to detect antibodies anti-*Toxoplasma gondii,* anti-*Leptospira* spp., anti*-Brucella canis* and anti-*Brucella abortus* in stray dogs housed in shelters at Umuarama city, Paraná, Brazil. In order to detect toxoplasmosis, indirect immunofluorescence assay (IFA) was performed, agglutination microscopic (MAT) test for leptospirosis and agar gel immunodiffusion (AGID) and buffered acidified antigen (BAA) tests for brucellosis.

**Results:**

Of the 175 serum samples analyzed, 70.85% were considered positive for toxoplasmosis by IFA, 20% by MAT for leptospirosis and 2.85% by AGID for *Brucella canis*.

**Conclusions:**

The serological results of this study showed that stray dogs housed at the private shelter are potential carriers of these three different zoonoses and contribute to the spread and maintenance of these etiologic agents in the urban area of Umuarama (PR), Brazil.

## Background

Leptospirosis is an infectious disease caused by bacteria of the *Leptospira* genus, which may affect diverse species of domestic and wild animals as well as humans. The transmission of this disease occurs by direct exposure to the urine or organs of infected animals, or indirectly when there is self-exposure to an environment contaminated with bacteria, such as standing water, wet soils, vegetation or fomites [1].

Canine Brucellosis is an infectious, chronic zoonotic disease whose etiologic agent is *Brucella* spp. The transmission occurs among animals by sexual contact between infected males and females or by exposure to an environment contaminated with secretions and/or infected placental membranes [2].

Toxoplasmosis is caused by *Toxoplasma gondii,* an obligate intracellular protozoan that utilizes domestic and wild cats as definitive hosts. Infection occurs by ingestion of oocysts spread in the environment, by cysts present in tissues of intermediate hosts and by transplacental transmission through tachyzoites [3].

Leptospirosis, toxoplasmosis and brucellosis are diseases with worldwide distribution. Among stray dogs, these zoonoses are facilitated by direct contact with other animals, by the habit of scavenging garbage and hunting in search of food, by drinking standing water, smelling other animals’ urine, licking female genitalia and by engaging in the sexual act itself [4-6].

In Brazil, different studies have shown the seroprevalence of leptospirosis, brucellosis and toxoplasmosis in stray dogs in the states of Minas Gerais, Santa Catarina, São Paulo, Rio de Janeiro, Bahia and Pará [5,7-12]. The prevalence rates reported in seroepidemiological studies of stray dogs contribute to elucidating the epidemiology of these diseases in different regions and provide data for the adoption of measures to prevent infection in humans.

Considering the absence of regional data and the importance of these agents in causing these diseases in humans, the objective of this study was to detect antibodies against *Leptospira* spp., *Brucella* spp. and *Toxoplasma gondii* in stray dogs housed in a shelter at Umuarama city, Paraná state, Brazil.

## Methods

### Sampling design

The town of Umuarama is located in the northwest region of the state of Paraná, Brazil. On the outskirts of the city, there is a private, philanthropic shelter maintained by staff and visitors; it collects dogs, cats and horses wandering around the city and forwards them for adoption.

The cross-sectional study included all dogs – regardless of age, gender or breed – residing in the shelter for the period March-May 2011. All dogs were pre-assessed by veterinarians regarding their nutritional status, hydration, body temperature, and abdominal discomfort. Exclusion criteria were dogs that had fever, dehydration, cachexia, anorexia, diarrhea, vomiting or any other clinical signs detectable at the time of blood collection.

### Collection of material

Up to 10 mL of blood was collected from each dog, by a veterinarian, via jugular venipuncture. Blood samples were identified and forwarded to the Laboratory of Preventive Veterinary Medicine at UNIPAR where they were centrifuged to obtain serum. Each sample was divided into three aliquots and stored in sterile flasks, identified and kept at - 20°C until use. Serological tests were performed in the laboratories the Leptospirosis and Zoonosis and Public Health of the Department of Preventive Veterinary Medicine at UNIPAR and at Londrina State University (UEL).

### Tests performed

To detect antibodies against anti-*Toxoplasma gondii*, the sera were submitted to indirect immunofluorescence assay (IFA) [13] employing commercial conjugate anti-Human IgG (Sigma®, USA). We used conjugates labeled with fluorescein isothiocyanate specific for dogs, positive and negative control sera and tachyzoites of the RH strain, kept by the Laboratory of Zoonoses and Public Health at the Londrina State University (PR) as antigens. Sera were tested in serial dilutions in base four to 1:4096, by which those presenting fluorescent tachyzoites at a titer ≥ 16 were considered reactive.

For detection of anti-*Leptospira* spp. antibodies, the samples were subjected to agglutination microscopic (MAT / AMT) test [14], using the following 22 reference serovars: Australis, Bratislava, Autumnalis, Butembo, Fortbragg, Castellonis, Bataviae, Canicola, Whitcombi, Cinoptery, Grippothyphosa, Hebdomadis, Copenhageni, Icterohaemorrhagiae, Panama, Pomona, Pyrogenes, Hardjo, Wolffi, Shermani, Tarassovi and Sentot – and kept at 28°C from 5 to 10 days in modified EMJH media (DIFCO®-USA) [15] in the Leptospirosis Laboratory, Londrina State University (UEL). Samples that exhibited at least 50% agglutinated *Leptospira* at a 1:100 dilution were considered reactive and then diluted at a two-to-one ratio to determine the maximum positive dilution. The analysis considered the most likely serovar the one that presented the highest agglutination titer whereas those that showed co-agglutination between serovars at higher dilution were considered reactive only to *Leptospira* spp. [16].

To detect antibodies, the anti-*Brucella canis* samples were submitted to an agar gel immunodiffusion (AGID) test using a commercial kit prepared with antigen (proteins and lipopolysaccharide) from *Brucella ovis*, REO198 sample, produced by the Paraná Institute of Technology - Tecpar. Samples were considered reactive when they showed a precipitin line between the well of the test serum and that of the antigen, as instructed by the manufacturer.

To detect anti-*Brucella abortus* antibodies, all sera were subjected to a screening test using the Buffered Acidified Antigen (BAA) test [17]. The antigen for BAA consists of an inactivated cell suspension of *Brucella abortus* 119–3 strain, pH 3.65 ± 0.05 at 8% concentration (Institute of Technology of Paraná-Tecpar). The test was considered positive when BAA macroscopic agglutination occurred.

## Results

We collected blood samples from 175 dogs of indeterminate breed, including 31 males and 144 females, aged between about 06 months and 13 years. Only eight dogs were excluded from the study due to their poor physical condition at the time of blood collection.

We detected 124/175 (70.85%) serum samples reactive for toxoplasmosis, 35/175 (20.00%) for leptospirosis and 05/175 (2.85%) for brucellosis.

For toxoplasmosis, 124 (70.85%) samples were considered reactive in IFA, 35 (28.23%) at the titer of 16, 58 (46.77%) at the 64 titer, 22 (17.75%) at the titer of 256, eight (6.45) at the 1024 titer and only one (0.80%) at the titer of 4096; the gender distribution is shown in Table 1.

**Table 1:** Prevalence the titers of antibodies detected in indirect immunofluorescence assay (IFA) for toxoplasmosis in 124 reactive serum samples of dogs housed in a private shelter at Umuarama municipality, Paraná, 2011

**Titers of antibodies**	**Prevalence**		**Total/%**
	**Female**	**Male**	
16	29	06	35 (28.23%)
64	46	10	56 (45.17%)
256	19	04	23 (18,55%)
1024	09	-	09 (07.25%)
4096	01	-	01 (0.80%)
Total	104 (83.87%)	20 (16.13%)	124 (100%)

In relation to leptospirosis, 35 (20.00%) samples were considered reactive in MAT, 24 (68.58%) had antibodies against one serotype: 10 (41.66%) samples positive for Canicola, nine (37.50%) for Bratislava, three (12.50%) for Tarassovi and two (8.34%) for Hardjo with titers of 100 to 800, while in 11 (31.42%) samples, antibodies against two serovars were detected simultaneously, thus enabling identification of the most likely serovar: Ten (90.90%) samples were positive for Canicola and one (9.10%) for Pyrogenes with titers between 100 to 12800 (Table 2).

**Table 2:** Most likely serovars and titers detected in agglutination microscopic (MAT) test for leptospirosis in 35 reactive serum samples from dogs housed in a private shelter at Umuarama municipality, Paraná, 2011

	**Serological titers**								
**Most likely serovars**	**100**	**200**	**400**	**800**	**1600**	**3200**	**12800**	**Total**	**(%)**
Canicola	05	03	03	02	04	02	01	20	57.14
Bratislava	09	-	-	-	-	-	-	09	25.71
Tarassovi	03	-	-	-	-	-	-	03	8.60
Hardjo	01	01	-	-	-	-	-	02	5.71
Pyrogenes	-	-	01	-	-	-	-	01	2.84
Total	18	04	04	02	04	02	01	35	100%

For brucellosis, five (2.85%) samples were considered reactive in AGID, compared with none in BAA. The prevalences of anti-*Toxoplasma gondii*, anti-*Leptospira* spp. and anti-*Brucella canis* antibodies in relation to gender of animals studied are shown in Table 3.

**Table 3:** **Prevalence of the antibodies anti- *Toxoplasma gondii *, anti-*Leptospira *spp. and anti- *Brucella canis *, by sex, in serum samples from dogs housed in a private shelter in the municipality of Umuarama, Paraná, 2011**

**Gender**	**Prevalence of antibodies**		
	**Anti-*Toxoplasma gondii***	**Anti-*Leptospira *spp.**	**Anti-*Brucella canis***
Female	104 (83.87%)	30 (85.72%)	03 (60%)
Male	20 (16.13%)	05 (14.28%)	02 (40%)
Total	124 (100%)	35 (100%)	05 (100%)

Of the 175 samples analyzed, reactive samples showed mixed infections: 14/175 (8%) positive for toxoplasmosis and leptospirosis; 03/175 (1.71%) for toxoplasmosis and brucellosis and 02/175 (1.14%) for toxoplasmosis, leptospirosis and brucellosis.

## Discussion and conclusion

Due to close contact with humans, different incubation periods and the possibility of asymptomatic cases in dogs with some zoonotic infections, dogs play an important role in the maintenance of different infectious and parasitic agents in the environment and their possible transmission to humans, a situation that constitutes a public health concern, especially in relation to stray dogs.

Different researchers have demonstrated the frequency of toxoplasmosis, leptospirosis and brucellosis in stray and domicilied dogs in different regions of Brazil, in order to characterize the epidemiology of these diseases at the study site [5,6,11]. However, the present work, in the city of Umuarama (PR), is the first time that these four diseases have been researched in stray dogs.

The finding that 70.85% of canines were considered positive for toxoplasmosis, exceeds the 65.55% and 26.00% seropositivity levels detected in stray dogs in Salvador (BA) [10] and Avaré (SP) [11], respectively. In the city of Avaré (SP) [11], from the 26.00% of samples reactive to *T. gondii*, ten (3.30%) presented an antibody titer of 16; 41 (13.70%) a titer of 64 and 27 (9.00%) a titer of 256, which are similar to the results of the present work; but no titers of 1024 or 4096 were detected. Yet in the state of São Paulo, in the city of Araçatuba [18], out of the 23.01% seroreactive to *T. gondii*, 44.00% presented a titer of 256, 12.00% a titer of 1024 and 4.00% a titer of 4096, levels higher than our current findings. The infection in dogs occurs mainly by ingestion of the infective form of the parasite. The oocysts of *Toxoplasma gondii,* after being eliminated in cat feces, sporulate in the environment and can contaminate food and water; cysts, in turn, are present in the tissues of intermediate hosts such as rodents, cattle, pigs, and others. Stray dogs are exposed to infection when seeking food by hunting small rodents or scavenging domestic waste, or when drinking pools of water on the streets.

It is known that toxoplasmosis is one of most prevalent zoonoses worldwide whose infection rates vary according to socioeconomic and cultural features of different localities. In Brazil, the varied disease rates described reflect its large territory while its high occurrence is influenced by climate and sanitation issues and even cultural habits [10,11]. In the state of Paraná, especially in the north, there are several studies about toxoplasmosis in humans, dogs, horses, cattle and pigs [19-22]. These results call for new research in order to elucidate the epidemiology of toxoplasmosis in northwestern Paraná.

From the standpoint of public health, the prevalence of stray dogs infected with *Toxoplasma gondii* indicates that the location studied provided ecological conditions to keep this parasite in its infective form for both canines and humans, with dogs in this case being useful as a sentinel for human toxoplasmosis [3].

Additional epidemiological research on the parasite would be critical for its control [12].

Seropositivity for leptospirosis in stray dogs in the present work was similar to the studies in Patos (PB) [7] and Londrina (PR) [6] and higher than the results in Itapema (SC) [8], which detected anti-*Leptospira* spp. antibodies at 20.00%, 21.21% and 10.50%, respectively. These serological results may have been influenced by differences in the prevalence of animal leptospirosis in the respective locations, besides reflecting the study period, which may have provided higher or lower probabilities of infection occurrence in stray dogs.

Research studies have reported [4,23] that leptospirosis is directly related to sanitation conditions, infrastructure deficiencies, and the presence of rodents in each region, a context that would explain the 20.00% prevalence in this study since stray dogs not only wander the streets scavenging garbage and possibly hunting rodents to feed themselves, but also might have been exposed to an environment contaminated with the infective bacterium.

In canine leptospirosis, animals can eliminate viable leptospires in urine intermittently over long periods, even when asymptomatic [24]. In the environment, the bacterium remains infective in humid and slightly alkaline places, such as in rainwater and mud puddles [23]. Making contact with these locations can lead to infection in dogs and humans. The prevalence detected in the present work, in stray dogs from different regions of the city, suggests the dispersal of leptospires in the Umuarama municipality (PR).

For the most prevalent serovars, Canicola, to which 57.14% of samples were reactive, has great epidemiological importance since the canines are considered its natural host and the presence of the bacterium in urine is common in asymptomatic dogs infected with this serovar [25]. The serovar Bratislava reacted in 37.50% of the samples and can be linked to close contact with infected horses, as this shelter also houses abandoned horses that were used as draft animals in the urban area of that city [26].

As to brucellosis, 2.85% of samples were found reactive by AGID for *Brucella canis*. This result is higher than those found in São João da Boa Vista (SP) [5] and lower than those in São Paulo (SP) [27], where the respective percentages detected in stray dogs were 0.80% and 7.50%. Transmission between dogs occurs through sexual contact. However, the infection can be established by the ingestion of food or water contaminated by *Brucella canis*[9]. The fact that these wandering dogs remain free on the streets and travel long distances allows dissemination of the agent in the city, and provides chances of infection to other animals and to humans through environmental contamination. The absence of antibodies to *Brucella abortus* in the BAA was justified by the non-exposure of stray dogs to potentially infected bovine and / or ingestion of raw milk, placental membranes or aborted fetuses contaminated, due to the permanence of stray dogs only in the urban area.

Thus, despite the low rate of brucellosis positivity in this study, the public health risk exists since we are studying stray dogs. The 2.85% presence of antibodies against *Brucella canis* in this study is sufficient to define the problem as epidemiologically important primarily due to the close contact of dogs with potential human owners, since this shelter houses animals are waiting for adoption.

The zoonotic feature of the *Brucella canis* infection was identified in 1969 [28], and the disease was subsequently characterized as occupational among laboratory workers or professions that have an established interaction with dogs, which arouses the attention of the employees of the private shelter [29]. These reasons demonstrate that the presence of anti-*Brucella* spp. is of fundamental importance in the local epidemiological study.

A concern that one should have in relation to stray dogs housed at the studied shelter is that toxoplasmosis, leptospirosis and brucellosis are diseases associated with workers who have frequent contact with possibly contaminated animals or their byproducts, which expose employees of the respective shelter to bacterial and parasitic infections. The awareness of the shelter employees studied, based on this research, is of great importance in the aspect of public health.

The serological results of this study showed that stray dogs housed at the private shelter are potential maintainers of these three different zoonoses and contribute to the spread and maintenance of these etiologic agents in the urban area of Umuarama (PR). Preventive measures such as neutering, adoption and responsible ownership campaigns would be key to reducing this problem studied in the city and elsewhere in the country.

## Ethics committee

This study was approved by the Ethics Committee for Animal Experiments (CEPEEA) of the University of Paraná (UNIPAR) under process number 20677/2011.

## Competing interests

The authors declare that there is no competing interest.

## Authors’ contributions

MKPD and DDG provided the idea; MKPD, PHM, GT and DB collection of biological samples; FMRLM, AB, DB and FE realization of serology; DDG, JCF, ITN and RLF analyzed the data; MKPD and DDG drafted the manuscript, and all authors read and approved the final manuscript.

## Financial source

Universidade Paranaense (UNIPAR – University of Paraná) and Coordination for Improvement of Higher Level Personnel (CAPES).
